# Inhibition of TRPV1 by SHP-1 in nociceptive primary sensory neurons is critical in PD-L1 analgesia

**DOI:** 10.1172/jci.insight.137386

**Published:** 2020-10-15

**Authors:** Ben-Long Liu, Qi-Lai Cao, Xin Zhao, Hui-Zhu Liu, Yu-Qiu Zhang

**Affiliations:** 1 State Key Laboratory of Medical Neurobiology and MOE Frontiers Center for Brain Science, Department of Translational Neuroscience, Jing’an District Centre Hospital of Shanghai, Institutes of Brain Science, Fudan University, Shanghai, China.; 2 Institutes of Integrative Medicine, Fudan University, Shanghai, China.

**Keywords:** Neuroscience, Pain, Signal transduction

## Abstract

Recently programmed death-ligand 1 (PD-L1) receptor PD-1 was found in dorsal root ganglion (DRG) neurons, and PD-L1 activates PD-1 to inhibit inflammatory and neuropathic pain by modulating neuronal excitability. However, the downstream signaling of PD-1 in sensory neurons remains unclear. Here, we show that PD-L1 activated Src homology 2 domain-containing tyrosine phosphatase-1 (SHP-1) to downregulate transient receptor potential vanilloid 1 (TRPV1) in DRG neurons and inhibit bone cancer pain in mice. Local injection of PD-L1 produced analgesia. PD-1 in DRG neurons colocalized with TRPV1 and SHP-1. PD-L1 induced the phosphorylation of SHP-1 in DRG TRPV1 neurons and inhibited TRPV1 currents. Loss of TRPV1 in mice abolished bone cancer–induced thermal hyperalgesia and PD-L1 analgesia. Conditioned deletion of SHP-1 in Na_V_1.8^+^ neurons aggravated bone cancer pain and diminished the inhibition of PD-L1 on TRPV1 currents and pain. Together, our findings suggest that PD-L1/PD-1 signaling suppresses bone cancer pain via inhibition of TRPV1 activity. Our results also suggest that SHP-1 in sensory neurons is an endogenous pain inhibitor and delays the development of bone cancer pain via suppressing TRPV1 function.

## Abstract 2

PD-L1/PD-1 signaling suppresses TRPV1 activity and alleviates pain-like behaviors via phosphorylation of SHP-1 in nociceptive primary sensory neurons in a mouse bone cancer model.

## Graphical abstract

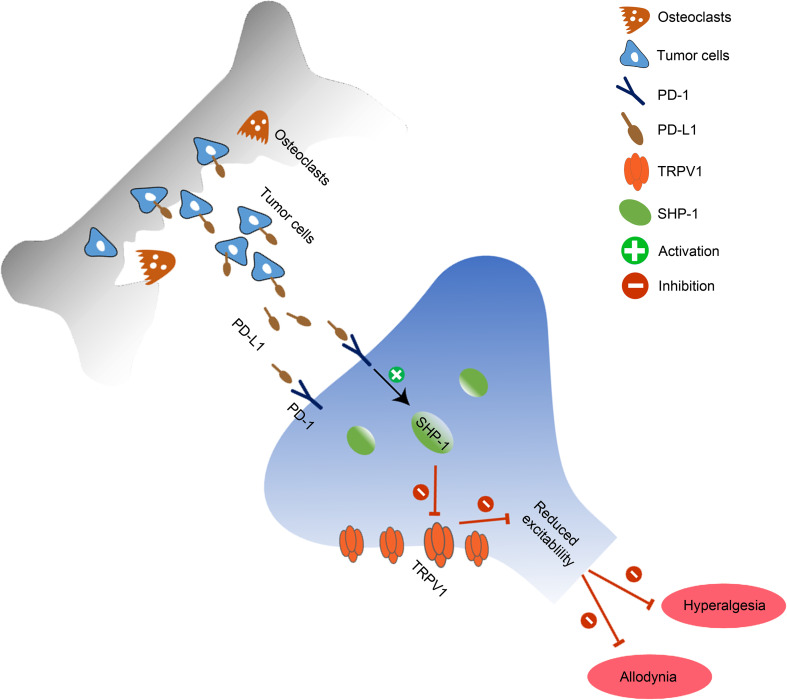

## Introduction

Cancer pain, reported by approximately 39.3%–66.4% of patients with cancer, severely reduces patients’ quality of life (1–3). One common type of cancer pain is bone cancer pain that occurs in patients with primary bone cancer and in patients with cancer that has metastasized to bone from distant sites, such as the breast, prostate, ovary, and lung (4). The neurochemistry and neurophysiology of cancer pain is complex. It is generally believed that cancer pain may involve a combination of inflammatory, neuropathic, ischemic, and compression mechanisms at multiple sites (5). During tumor growth and invasion, multiple algogenic substances released from cancer cells, osteoblasts, osteoclasts, and nerve endings sensitize and activate primary afferent nociceptors in the cancer microenvironment (6, 7). One important concept that has emerged over the past decade is that sensitization of transient receptor potential vanilloid 1 (TRPV1) plays an important role in driving bone cancer pain (5, 8–10). TRPV1 is a ligand-gated nonselective cation channel, which is mainly expressed in small-diameter (<25 μm) DRG neurons. It can be activated by a series of physical and chemical signals, including noxious heat (>43°C), extracellular protons, and vanilloid capsaicin (11). In a rat bone cancer model using Walker 256 mammary gland carcinoma cells, we observed that TRPV1 expression level and capsaicin-induced TRPV1 currents are increased in ipsilateral DRG neurons with tumor inoculation in bone, and blockade of TRPV1 reduces bone cancer pain (10). Consistently, an earlier study from Ghilardi et al. found that TRPV1-knockout mice show a significant reduction in ongoing and movement-evoked nocifensive behaviors in bone cancer mice (12).

Although the cancer microenvironment can produce and secrete a variety of mediators, current studies almost always focus on pronocicepetive actions of cancer-produced mediators (5). However, some cancers such as melanoma, as well as early-stage cancers, are not painful or cause minimal pain (13, 14). Recently, a cooperative study from Ji’s laboratory and our laboratory revealed the antinociceptive effects of programmed death-ligand 1 (PD-L1) (15). Since many cancers express PD-L1 (16, 17), it is conceivable that tumors could bidirectionally modulate pain through positive factors mentioned above and negative factors like PD-L1.

PD-L1/PD-1 can recruit Src homology 2 domain-containing tyrosine phosphatase-1 (SHP-1) and SHP-2 to mediate the biological actions of PD-L1 in immune cells (18, 19). Our previous study has demonstrated that PD-L1 in DRG nociceptive neurons inhibits sodium channels, activates TREK2 K^+^ channels, and suppresses neuronal excitability by phosphorylation of the tyrosine phosphatase SHP-1 (15). Tyrosine phosphorylation is also important for TRPV1 activation (20). SHP-1 has been reported to alleviate CFA-induced inflammatory pain through dephosphorylation of TRPV1 in DRG neurons of rats (21). However, whether PD-L1/PD-1 regulates TRPV1 remains unknown. In the present study, we investigate the role of PD-L1/PD-1 in mouse bone cancer pain by Lewis lung carcinoma (LLC) cell inoculation and address whether PD-L1 modulates TRPV1 in DRG nociceptive neurons from control mice and mice with cancer pain. Moreover, we try to reveal the effects of deleting SHP-1 in Na_V_1.8^+^ primary sensory neurons on PD-L1/PD-1 regulating TRPV1 activity and bone cancer pain.

## Results

### Intrafemur inoculation of LLC induces bone cancer pain in mice.

**Figure 1:**
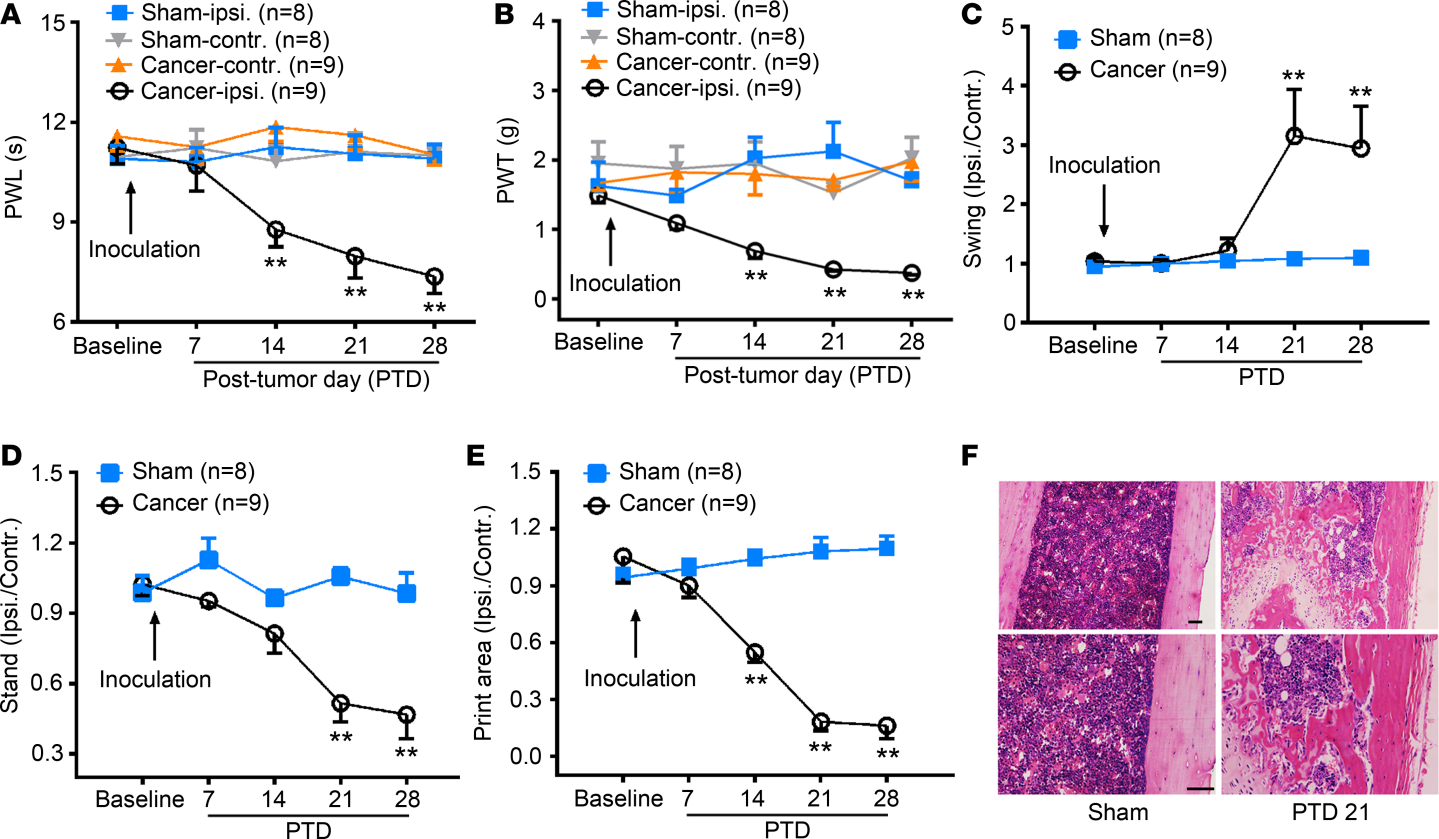
Bone cancer induces pain-like behaviors and bone destruction in mice. (**A** and **B**) Intrafemur inoculation with Lewis lung carcinoma cells (LLC, 1 × 10^6^) induces significant thermal hyperalgesia (**A**) and mechanical allodynia (**B**) in the ipsilateral hind paw. **P < 0.01 versus sham control; 2-way RM ANOVA followed by post hoc Student-Newman-Keuls test; *n* = 8 sham and 9 cancer (mice). Ipsi, ipsilateral; Contr, contralateral; PWL, paw withdrawal latency; PWT, paw withdrawal threshold. (**C**–**E**) CatWalk gait analysis showing increased swing phase on PTDs 21 and 28 (**C**), as well as decreased stand phase on PTDs 21 and 28 (**D**) and print area on PTDs 14–28 (**E**) in bone cancer mice. Data were calculated as the percentage of ipsilateral/contralateral hind paw. **P < 0.01 versus sham ones; 2-way RM ANOVA followed by post hoc Student-Newman-Keuls test; *n* = 8 sham and 9 cancer (mice). (**F**) Histopathological sections (hematoxylin and eosin stain) showing that the bone marrow was replaced by invading tumor cells with medullary bone loss and femur bone destruction on PTD 21. Scale bar: 100 μm.

After intrafemur inoculation of LLC cells, mice developed mechanical allodynia and thermal hyperalgesia by posttumor day (PTD) 14 that persisted for at least 28 days in the ipsilateral hind paw (Figure 1, A and B, 2-way repeated measures [RM] ANOVA, treatments: *F*_[3, 30]_ = 20.90, *P* < 0.001 for PWL; *F*_[3, 30]_ = 24.31, *P* < 0.001 for PWT; treatment × time: *F*_[12, 120]_ = 3.10, *P* < 0.001 for PWL; *F*_[12, 120]_ = 1.97, *P* = 0.03 for PWT). CatWalk gait analysis can rapidly quantify several gait parameters, including the mean paw print area and stand and swing phases of the hind paw, which have been linked to mechanical allodynia and spontaneous pain in chronic pain (22–24). As shown in Figure 1, C–E, bone cancer mice exhibited a significant increase in swing phase (i.e., the duration of the paw not touching the glass plate) of the affected paw on PTDs 21 and 28 (2-way RM ANOVA, treatments: *F*_[1, 15]_ = 14.52, *P* < 0.001; treatment × time: *F*_[4, 60]_ = 4.40, *P* < 0.001). The stand phase (i.e., the duration of the paw touching the glass plate) and print area (i.e., the surface area of the complete paw print) were decreased in the affected paw of bone cancer mice on PTDs 14–28 (Figure 1, D and E, 2-way RM ANOVA, treatments: *F*_[1, 15]_ = 35.35, *P* < 0.001 for stand; *F*_[1, 15]_ = 92.36, *P* < 0.001 for print area), suggesting that the bone cancer mice avoided carrying weight on their affected hind limb when walking. Given that pain-like behaviors steadily appeared by PTD 21, we selected PTD 21 to perform the following experiments. The hematoxylin and eosin–stained femur sections showed both tumor growth and bone destruction with medullary bone loss on PTD 21 (Figure 1F).

### PD-L1 inhibits mouse bone cancer pain.

**Figure 2:**
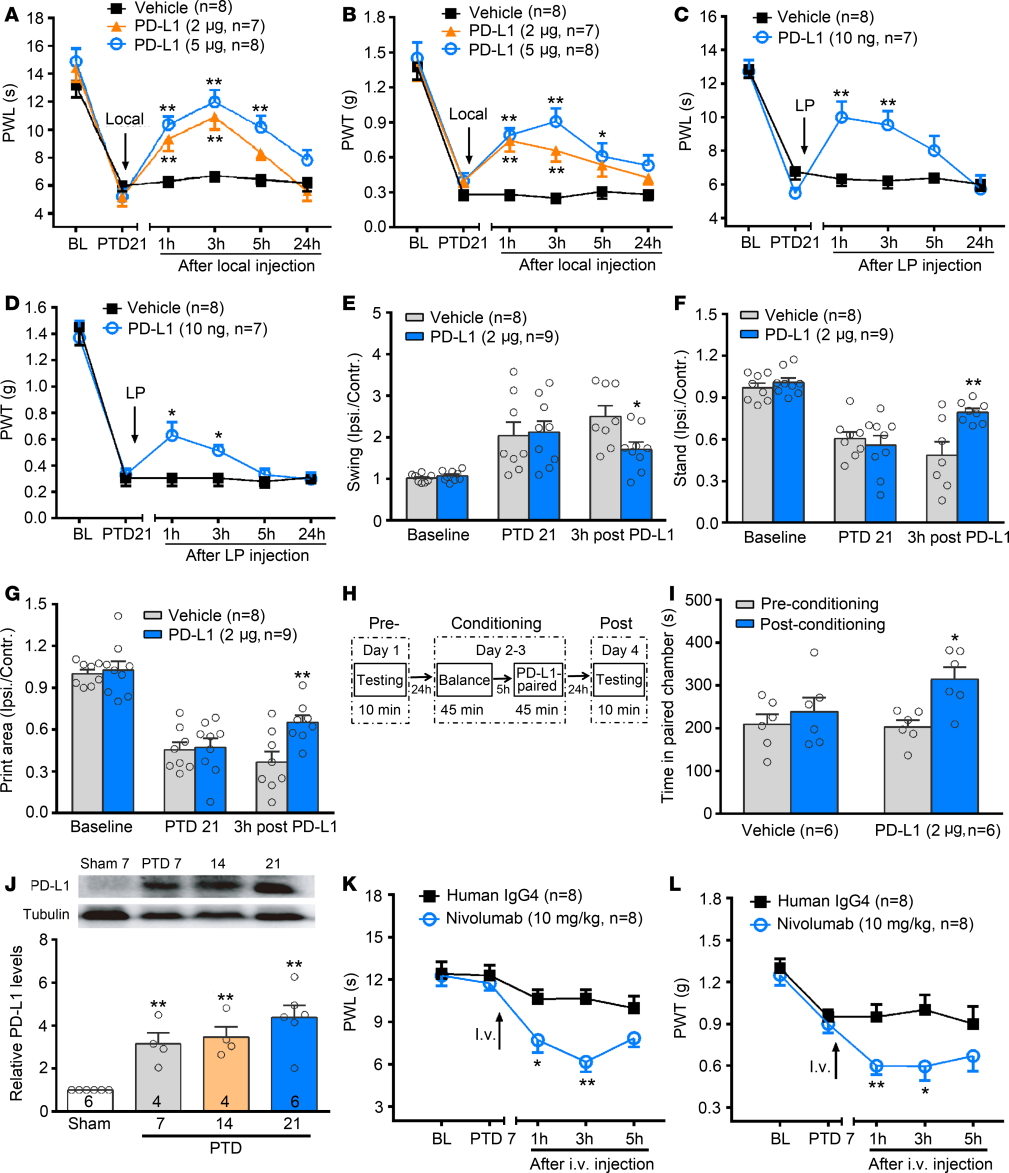
Involvement of PD-L1/PD-1 in mouse bone cancer pain. (**A**–**D**) Local (**A** and **B**) and lumbar puncture (LP) injection (**C** and **D**) of PD-L1 alleviates bone cancer–induced thermal hyperalgesia (**A** and **C**) and mechanical allodynia (**B** and **D**) on PTD 21. *P < 0.05, **P < 0.01 versus vehicle control; 2-way RM ANOVA followed by post hoc Student-Newman-Keuls test; *n* = 8 vehicle, 7 PD-L1 2 μg, 8 PD-L1 5 μg for **A** and **B**, *n* = 8 vehicle and 7 PD-L1 10 ng for **C** and **D** (mice). (**E**–**G**) Local injection of PD-L1 ameliorates bone cancer–induced changes in swing (**E**), stand (**F**), and print area (**G**) on PTD 21 by CatWalk gait analysis. *P < 0.05, **P < 0.01 versus vehicle control; 2-way RM ANOVA followed by post hoc Student-Newman-Keuls test; *n* = 8 vehicle and 9 PD-L1 2 μg (mice). (**H**) Schematic of the protocol for conditioned place preference (CPP). (**I**) Local injection of PD-L1 induces CPP. *P < 0.05 versus preconditioning; 2-way ANOVA followed by post hoc Student-Newman-Keuls test; *n* = 6 vehicle and 6 PD-L1 (mice). (**J**) Western blot analysis reveals an increase in the level of PD-L1 in the affected bone after tumor inoculation. PD-L1 level is expressed as fold increase compared with sham controls at each corresponding time point. **P < 0.01 versus sham control, 1-way ANOVA followed by post hoc Student-Newman-Keuls test; *n* = 6 sham mice, 4 PTD 7 mice, 4 PTD 14 mice, 6 PTD 21 mice. (**K** and **L**) Neutralization of PD-1 by nivolumab (anti–PD-1 antibody, 10 mg/kg, i.v.) induces thermal hyperalgesia (**K**) and mechanical allodynia (**L**) in the early phase of bone cancer (PTD 7). *P < 0.05, **P < 0.01 versus IgG control; 2-way RM ANOVA followed by post hoc Student-Newman-Keuls test; *n* = 8 human IgG4 and 8 nivolumab (mice).

Our previous study found that peripheral administration of PD-L1 produces an analgesic effect in naive and formalin-inflamed animals (15). Here, we further addressed whether PD-L1 antagonizes bone cancer–induced pain. Local injection (deep tissue around tumor bone) of PD-L1 (2 or 5 μg) (15) in the PTD 21 mice significantly suppressed bone cancer–induced thermal hyperalgesia and mechanical allodynia (Figure 2, A and B, 2-way RM ANOVA, treatments: *F*_[2, 20]_ = 21.59, *P* < 0.001, treatment × time: *F*_[10, 100]_ = 3.30, *P* < 0.001 for PWL; treatments: *F*_[2, 20]_ = 21.39, *P* < 0.001, treatment × time: *F*_[10, 100]_ = 1.95, *P* = 0.04 for PWT). Consistently, bone cancer–induced thermal hyperalgesia and mechanical allodynia were also attenuated by lumbar puncture (LP) injection of PD-L1 (10 ng, Figure 2, C and D). It has been reported that drugs can be predominantly delivered into DRG neurons by direct LP (10, 25). Also, CatWalk gait analysis revealed that bone cancer–induced pain-like behaviors were partially reversed by local PD-L1 injection (Figure 2, E–G). Moreover, using a conditioned place preference (CPP) test, an operant measurement of ongoing pain (26–28), we observed that PD-L1 (2 μg) treatment resulted in a marked CPP (Figure 2, H and I). When PD-L1 treatment was paired with a particular chamber in the place-conditioning apparatus, mice spent more time in the chamber on the postconditioning day compared with the preconditioning day (Figure 2I, 2-way ANOVA, pre versus post: *F*_[1, 10]_ = 7.27, *P* = 0.014).

PD-L1 has been detected in LLC cell lines (16) and gliomas (17). We also found a high-level expression of PD-L1 in melanoma (15). We therefore examined PD-L1 expression level in tumor-bearing bone in the present study. The level of PD-L1 protein was obviously upregulated in tumor-bearing bone from PTDs 7 to 21 after carcinoma inoculation (Figure 2J, 1-way ANOVA, *F*_[3, 15]_ = 10.78, *P* < 0.001). Consistent with our previous report (15), PD-L1’s receptor, PD-1, was detected on DRG neurons by in situ hybridization, immunohistochemistry, reverse transcription PCR (RT-PCR) and Western blot (Supplemental Figure 1, A–F; supplemental material available online with this article; https://doi.org/10.1172/jci.insight.137386DS1). No significant change of PD-1 expression was found in the DRG ipsilateral to the tumor-bearing hind limb during the development of bone cancer (Supplemental Figure 1F). We have demonstrated that peripheral local injection of soluble PD-1 to neutralize endogenous PD-L1 induces a transient mechanical allodynia in naive mice (15). Thus, increased peripheral PD-L1 in the early phase of bone cancer may mask hyperalgesia and allodynia development. As expected, on PTD 7 when the pain-like behaviors had not yet occurred, neutralization of PD-1 by nivolumab (anti–PD-1 antibody, 10 mg/kg, i.v.) directly induced thermal hyperalgesia and mechanical allodynia (Figure 2, K and L, 2-way RM ANOVA, treatments: *F*_[1, 14]_ = 19.84, *P* < 0.001 for PWL; *F*_[1, 14]_ = 14.96, *P* < 0.001 for PWT).

### TRPV1 is involved in PD-L1–induced inhibition of bone cancer pain.

**Figure 3:**
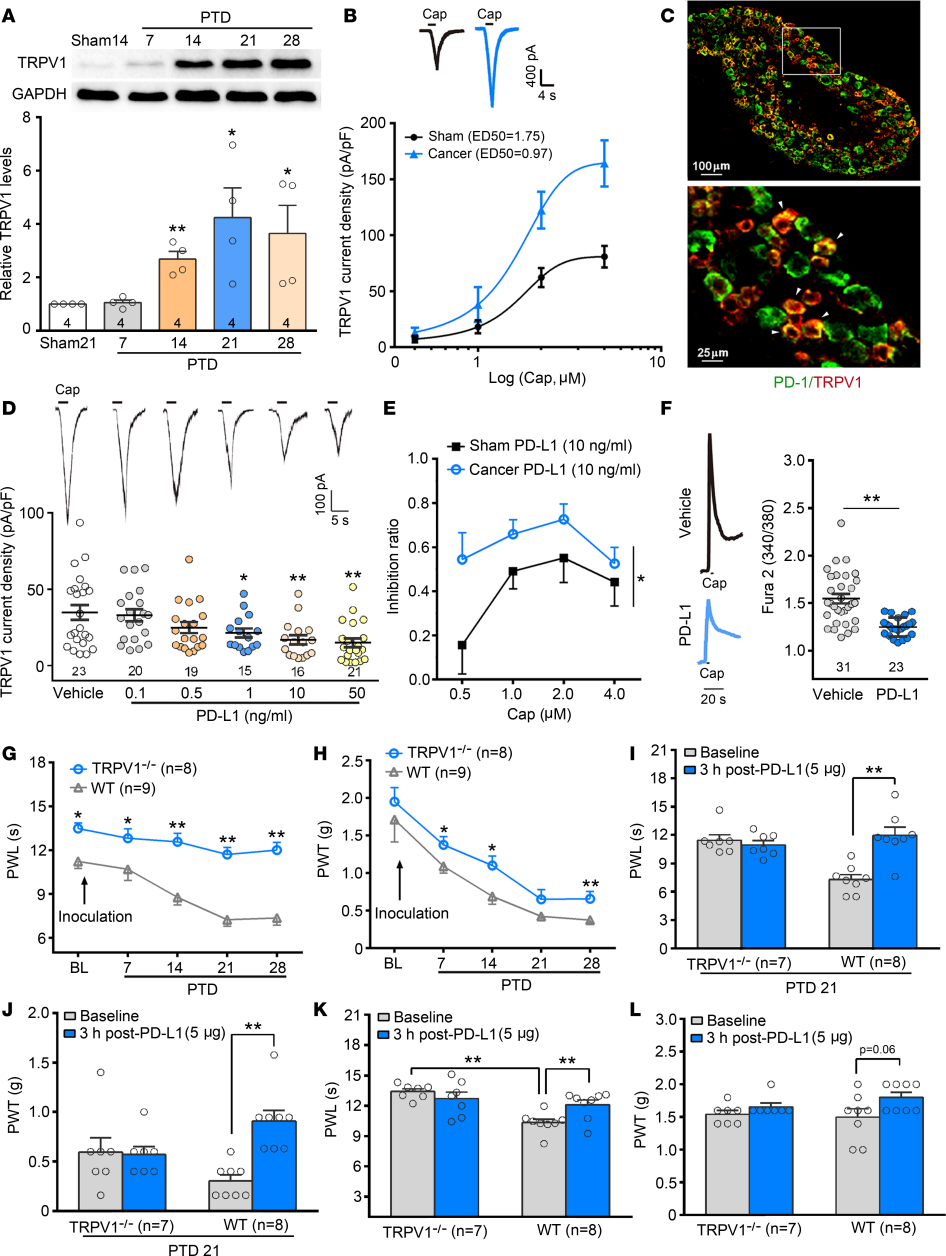
Dynamic changes in TRPV1 in DRG neurons after tumor inoculation and effects of PD-L1 on TRPV1 function. (**A**) Western blot analysis reveals a significant increase in the level of TRPV1 in L_3_–L_5_ DRGs ipsilateral to the tumor-bearing bone after tumor inoculation. *P < 0.05, **P < 0.01 versus sham control; 1-way ANOVA followed by post hoc Student-Newman-Keuls test; *n* = 4 for all the groups (mice). (**B**) Capsaicin dose-response curves are left-shifted in small-diameter (<25 μm) DRG neurons from PTD 21 mice compared with those from sham ones. Insert represents 1.5 μM capsaicin–evoked TRPV1 current traces in individual DRG neurons of sham and PTD 21 mice. (**C**) Double immunofluorescence reveals colocalization of PD-1 with TRPV1 in L_4_ DRG neurons. Scale bar: 100 μm (upper), 25 μm (bottom). (**D**) Different doses of PD-L1 suppresses TRPV1 current density (pA/pF) in DRG neurons. Insert represents 1.5 μM capsaicin–evoked TRPV1 current traces in individual DRG neurons after different doses of PD-L1 treatment. *P < 0.05, **P < 0.01 versus vehicle control; 1-way ANOVA followed by post hoc Student-Newman-Keuls test; *n* = 23 vehicle, 20 PD-L1 0.1 ng/mL, 19 PD-L1 0.5 ng/mL, 15 PD-L1 1 ng/mL, 16 PD-L1 10 ng/mL, and 21 PD-L1 50 ng/mL (cells). (**E**) The inhibition ratio of PD-L1 (10 ng/mL) on TRPV1 currents by different concentrations of capsaicin. *P < 0.05 versus sham control; 2-way RM ANOVA followed by post hoc Student-Newman-Keuls test; *n* = 9 capsaicin 0.5 μM, 10 capsaicin 1 μM, 6 capsaicin 2 μM, and 16 capsaicin 4 μM from sham mice; *n* = 10 capsaicin 0.5 μM, 9 capsaicin 1 μM, 14 capsaicin 2 μM, and 10 capsaicin 4 μM from cancer mice (cells). (**F**) Traces (left) of calcium imaging from individual DRG neurons of bone cancer and sham mice challenged by capsaicin application. Scatter plot (right) showing that PD-L1 significantly attenuates capsaicin-induced increase in [Ca^2+^]_i_. **P < 0.01 versus vehicle control; 2-tailed Student’s *t* test; *n* = 23 vehicle and 31 PD-L1 (cells). (**G**) TRPV1^–/–^ mice fail to develop thermal hyperalgesia in tumor-bearing limbs. *P < 0.05, **P < 0.01 versus WT mice; 2-way RM ANOVA followed by post hoc Student-Newman-Keuls test; *n* = 8 TRPV1^–/–^ and 9 WT (mice). (**H**) TRPV1^–/–^ mice develop lighter mechanical allodynia than the WT mice following the tumor inoculation. *P < 0.05, **P < 0.01 versus WT mice; 2-way RM ANOVA followed by post hoc Student-Newman-Keuls test; *n* = 8 TRPV1^–/–^ and 9 WT (mice). (**I** and **J**) PD-L1 inhibits bone cancer–induced thermal hyperalgesia (**I**) and mechanical allodynia (**J**) in WT mice but not in TRPV1^–/–^ mice on PTD 21. **P< 0.01; paired Student’s *t* test; *n* = 7 TRPV1^–/–^ and 8 WT (mice). (**K** and **L**) Intraplantar injection of PD-L1 induces a significant increase in PWL (**K**) and an increasing tendency in PWT (**L**) in naive WT but not TRPV1^–/–^ mice. **P < 0.01; 2-tailed paired Student’s *t* test; *n* = 7 TRPV1^–/–^ and 8 WT (mice).

The tumor-induced local environment acidification can activate TRPV1 at normal body temperatures (29, 30). TRPV1 has been proved an important ion channel closely related to peripheral sensitization of pain (31–33). Western blot analysis showed significant upregulation of TRPV1 level in DRG tissues ipsilateral to the tumor-bearing bone from PTDs 14 to 28 (Figure 3A, 1-way ANOVA, *F*_[4, 14]_ = 3.83, *P* = 0.02). Consistent with our previous study in rats, whole-cell patch clamp recordings showed that capsaicin-induced (0.5–4.0 μM) TRPV1 currents in small-diameter (<25 μm) DRG neurons of bone cancer mice significantly increased as compared with those of sham ones. The dose-response curve of TRPV1 currents was left-shifted on PTD 21 (Figure 3B), suggesting the sensitization of TRPV1 in bone cancer mice. Next, we examined whether PD-L1–induced inhibition of bone cancer pain is partially achieved by modulating TRPV1. Double immunostaining showed coexpression of PD-1 and TRPV1 in DRG small-diameter neurons (Figure 3C). Patch clamp recordings showed that PD-L1 (0.1–50 ng/mL, for 30 minutes) dose-dependently suppressed TRPV1 currents (Figure 3D, 1-way ANOVA, *F*_[5, 108]_ = 4.87, *P* < 0.001). The inhibition of PD-L1 (10 ng/mL) on TRPV1 currents by different doses of capsaicin was greater in bone cancer mice than that in sham mice (Figure 3E, 2-way RM ANOVA, treatments: *F*_[1, 82]_ = 4.11, *P* = 0.04).

Analogously, capsaicin-induced [Ca^2+^]_i_ increase was significantly blocked by pretreatment with 10 ng/mL of PD-L1 (Figure 3F, 2-tailed Student’s *t* test, *t*_[52]_ = 4.94, *P* < 0.001). To further clarify the role of TRPV1 in PD-L1–induced inhibition, we examined the effects of PD-L1 on bone cancer pain in TRPV1-knockout mice. Although LLC cell inoculation resulted in a similar tumor growth in TRPV1-knockout mice (B6.129X1-Trpv1^tm1JμL/^J; hereafter TRPV1-KO or TRPV1^–/–^) and WT mice, TRPV1^–/–^ mice failed to develop thermal hyperalgesia in the affected limb (Figure 3G). TRPV1^–/–^ mice could still develop mechanical allodynia following the tumor inoculation, but the bone cancer–induced mechanical allodynia in TRPV1^–/–^ mice was less than in WT mice (Figure 3H, 2-way RM ANOVA, treatment: *F*_[1, 15]_ = 10.19, *P* = 0.002). Also, CatWalk gait analysis showed lower pain-like behaviors in TRPV1^–/–^ mice than in WT ones (Supplemental Figure 2). Neither PWL nor bone cancer–induced mechanical allodynia was changed by local injection of PD-L1 (5 μg) in tumor-bearing TRPV1^–/–^ mice (Figure 3, I and J). In addition, we examined the effect of PD-L1 on normal WT and TRPV1^–/–^ mice’s basal response threshold to thermal and mechanical stimuli. Intraplantar injection of PD-L1 (5 μg) significantly increased PWL in WT but not TRPV1^–/–^ mice (Figure 3, K and L).

### PD-L1 inhibits TRPV1 function via SHP-1.

**Figure 4:**
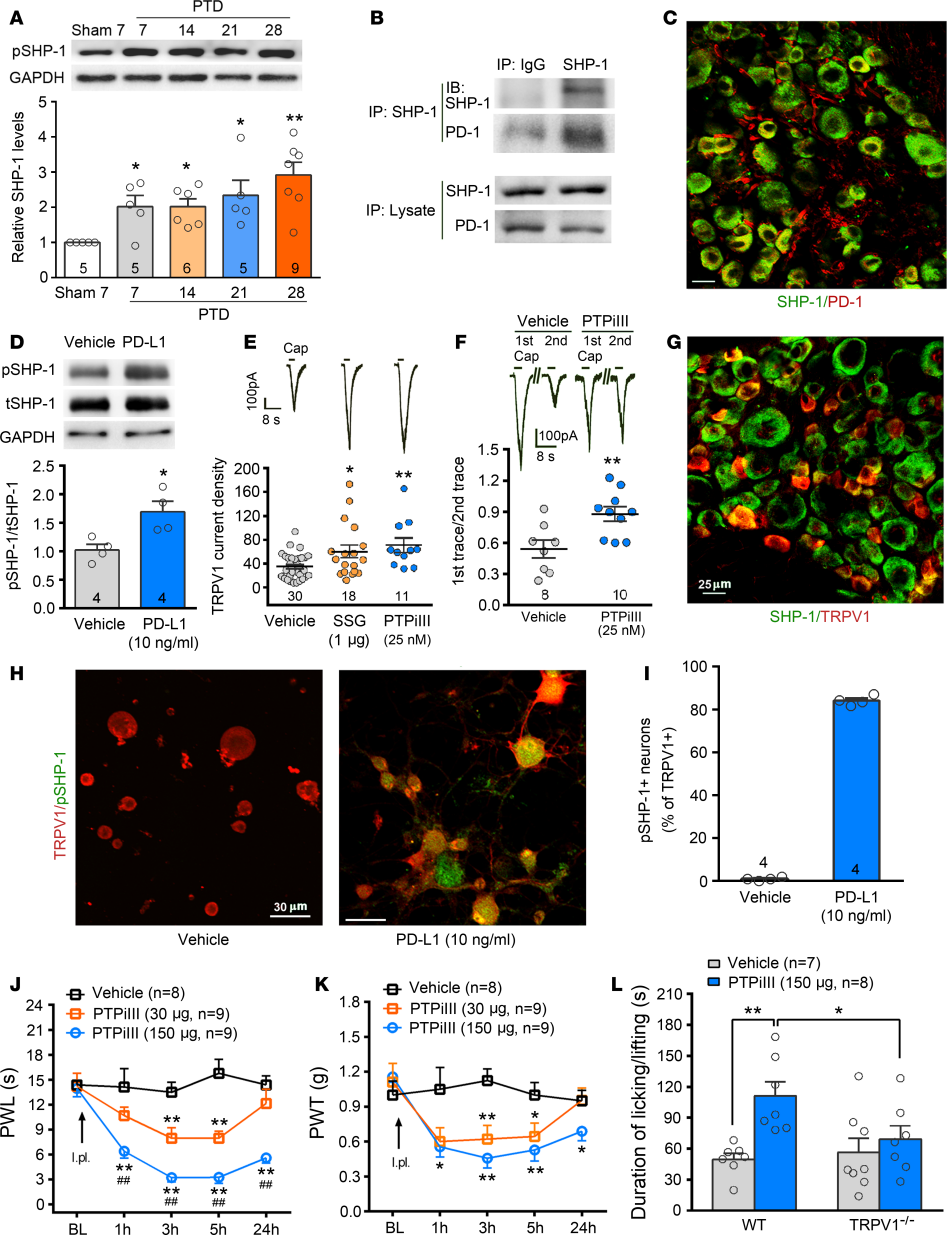
Inhibition of SHP-1 sensitizes TRPV1 and facilitates pain-like behaviors. (**A**) Western blot analysis showing an increase in the level of phosphorylated SHP-1 (pSHP-1) in L_3_–L_5_ DRGs ipsilateral to the tumor-bearing bone after tumor inoculation. *P < 0.05, **P < 0.01 versus sham control; 1-way ANOVA followed by post hoc Student-Newman-Keuls test; *n* = 5 sham treatment day 7, 5 PTD 7, 6 PTD 14, 5 PTD 21, and 9 PTD 28 (mice). (**B**) Coimmunoprecipitation displays that PD-1 and SHP-1 are captured with anti–SHP-1 in L_3_–L_5_ DRGs. Normal rabbit IgG immunoprecipitation was performed as the negative control. Three individual trials were performed to repeat the result. (**C**) Double immunofluorescent staining detected colocalization of SHP-1 and PD-1 in the L_4_ DRG. Scale bar: 25 μm. (**D**) PD-L1 (50 ng/mL, 30 minutes) treatment increases tyrosine phosphorylation of SHP-1 in cultured DRG neurons. *P < 0.05 versus control; 2-tailed Student’s *t* test; *n* = 4 vehicle and 4 PD-L1 (mice). (**E**) Whole-cell patch clamp recording showing that specific SHP-1 inhibitor SSG (1 μM) or PTPiIII (25 nM) increases 1.5 μM capsaicin–evoked TRPV1 current density in DRG small-diameter neurons. Insert represents TRPV1 current traces in individual DRG neurons. *P < 0.05, **P < 0.01 versus vehicle control; 1-way ANOVA followed by post hoc Student-Newman-Keuls test; *n* = 30 vehicle, 18 SSG, and 11 PTPiIII (cells). (**F**) SHP-1 inhibitor PTPiIII attenuates the repeated capsaicin-induced desensitization, showing increased ratio of second trace peak amplitude to first trace peak amplitude. Insert represents TRPV1 current traces in vehicle- and PTPiIII-treated neurons. **P < 0.01 versus vehicle control; 2-tailed Student’s *t* test; *n* = 8 vehicle and 10 PTPiIII (cells). (**G**) Double immunofluorescent staining detected colocalization of SHP-1 and TRPV1 in the L_4_ DRG. Scale bar: 25 μm. (**H** and **I**) Immunocytochemistry double staining of TRPV1 and pSHP-1 in cultured DRG neurons treated by vehicle and PD-L1 (10 ng/mL, 30 minutes). After incubation in PD-L1 for 30 minutes, pSHP-1 was detected in most TRPV1-positive neurons; *n* = 4 vehicle and 4 PD-L1. Scale bar: 30 μm. (**J** and **K**) Intraplantar injection of SHP-1 inhibitor PTPiIII (30 and 150 μg) directly induces thermal hyperalgesia (**J**) and mechanical allodynia (**K**). *P < 0.05, **P < 0.01 versus vehicle control; ^##^*P* < 0.01 versus 30 μg PTPiIII, 2-way RM ANOVA followed by post hoc Student-Newman-Keuls test; *n* = 8 vehicle, 9 PTPiIII 30 μg, and 9 PTPiIII 150 μg (mice). (**L**) Intraplantar injection of SHP-1 inhibitor PTPiIII (150 μg) evokes spontaneous pain that manifested by licking and flinching within 3 minutes after the injection in naive WT mice and TRPV1^–/–^ mice. *P < 0.05, **P < 0.01; 1-way ANOVA followed by post hoc Student-Newman-Keuls test; *n* = 7 vehicle and 8 PTPiIII (mice).

Recently, we revealed that PD-L1 modulates TREK2 potassium channels via SHP-1 in mouse DRG neurons (15). In this study, we further investigated the role of SHP-1 in PD-L1 modulating TRPV1. Western blot showed that SHP-1 protein expression was significantly increased from PTDs 7 to 28 in the DRG tissues ipsilateral to the tumor-bearing limb (Figure 4A, 1-way ANOVA, *F*_[4, 23]_ = 4.33, *P* < 0.01). Coimmunoprecipitation displayed that SHP-1 protein was immunoprecipitated by the anti–SHP-1 antibody together with the PD-1 protein, indicating their interaction (Figure 4B). Double immunofluorescent staining detected colocalization of SHP-1 and PD-1 (Figure 4C). In cultured DRG neurons, PD-L1 (10 ng/mL, 30 minutes) treatment upregulated phosphorylated SHP-1 (p–SHP-1) but did not affect total SHP-1 (Figure 4D, 2-tailed Student’s *t* test, *t*_[6]_ = 3.22, *P* = 0.02). We next examined whether SHP-1 modulates TRPV1 current in mouse DRG neurons. The specific SHP-1 inhibitor sodium stibogluconate (SSG) (1 μM) or PTP inhibitor iIII (PTPiIII) (25 nM) in the intracellular solution significantly increased the TRPV1 current density in DRG small-diameter neurons (Figure 4E, 1-way ANOVA, *F*_[2, 56]_ = 5.81, *P* < 0.01). As reported previously (10), repeated applications of 1 μM capsaicin (3 seconds, interval of 60 seconds) produced a desensitization in the responses in the neurons (Figure 4F). PTPiIII markedly prevented the repeated capsaicin-induced desensitization (Figure 4F, 2-tailed Student’s *t* test, *t*_[16]_ = 3.11, *P* < 0.01). Double immunofluorescent staining showed that SHP-1 was colocalized with TRPV1 (Figure 4G). Furthermore, PD-L1 (10 ng/mL, 30 minutes) in cultured DRG neurons produced a robust p–SHP-1 expression in more than 80% of TRPV1-positive neurons, providing a cytological basis for rapid modulation of TRPV1 by SHP-1 (Figure 4, H and I). Behavioral data showed that intraplantar injection (i.pl.) of SHP-1 inhibitor PTPiIII (30 or 150 μg, 20 μL) evoked robust thermal hyperalgesia and mechanical allodynia in naive mice (Figure 4, J and K, 2-way RM ANOVA, treatment: *F*_[2, 23]_ = 53.85, *P* < 0.001 for PWL; *F*_[2, 23]_ = 12.26, *P* < 0.01 for PWT). I.pl. PTPiIII (150 μg) also induced spontaneous pain, manifested by licking and flinching in treated paws within 3 minutes after the injection in naive WT mice. However, PTPiIII failed to induce spontaneous pain in TRPV1-KO mice (Figure 4L).

### Inhibitory effect of PD-L1 on DRG neurons decreases in SHP-1–conditional KO mice.

**Figure 5:**
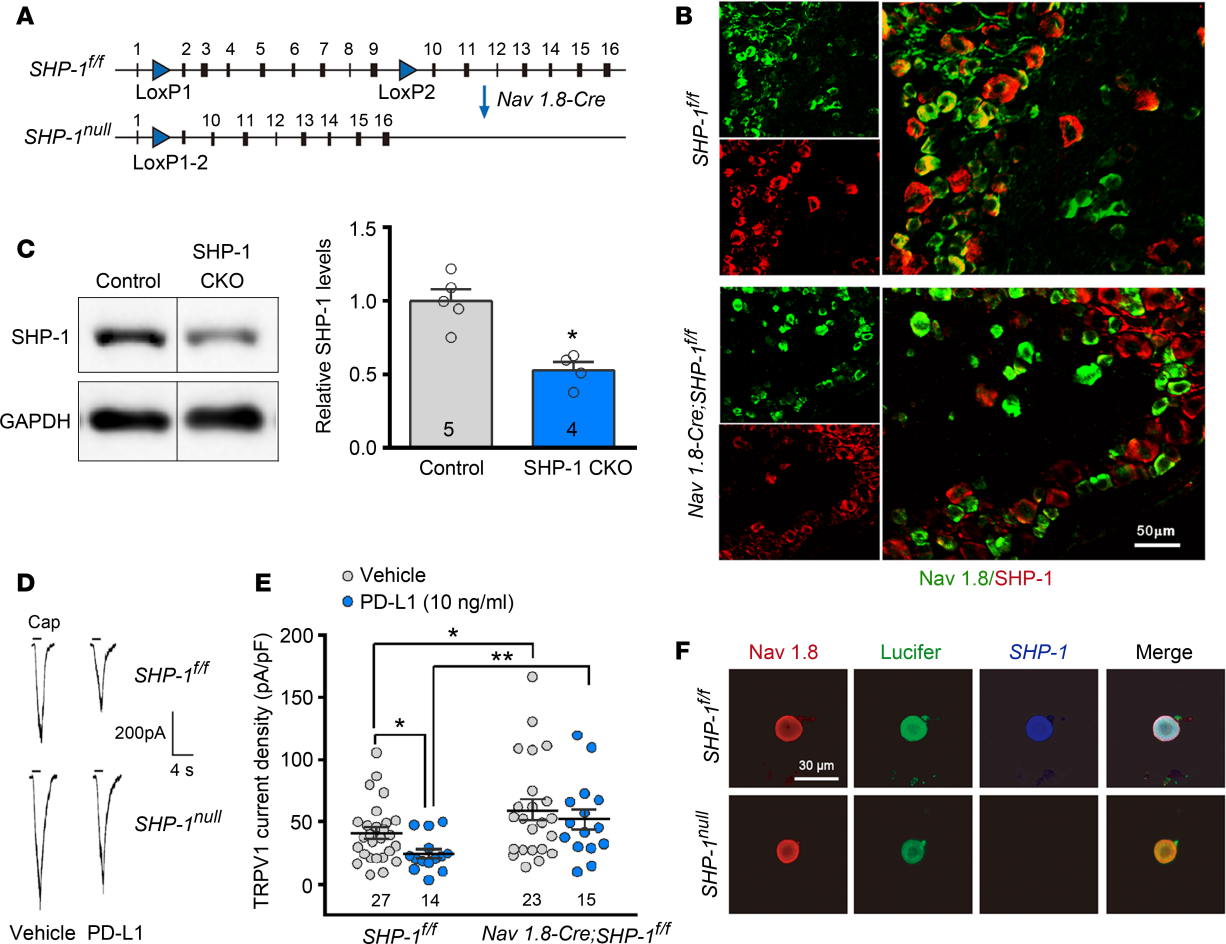
PD-L1 inhibits TRPV1 via SHP-1. (**A**) Schematic illustration of SHP-1–CKO mice by Cre-Lox system crossing *SHP-1*^fl/fl^ mice with *Na_V_1.8-Cre* mice. (**B**) Double immunofluorescence reveals colocalization of Nav1.8 and SHP-1 in the L_4_ DRG of control mice but no double staining signal of SHP-1 and Na_V_1.8 in the L_4_ DRG of SHP-1 CKO (bottom). Scale bar: 50 μm. (**C**) Western blot analysis showing a significant reduction of SHP-1 in the L_3_–L_5_ DRGs of CKO mice. *P < 0.05 versus littermate controls, 2-tailed Student’s *t* test; *n* = 5 control and 4 SHP-1–CKO (mice). (**D**) Traces showing that PD-L1 suppresses 1.5 μM capsaicin–evoked TRPV1 currents in DRG neurons of littermate control mice (upper) but not in SHP-1–CKO mice (bottom). (**E**) Quantification of changes in TRPV1 current density in DRG neurons of SHP-1–CKO and littermate control mice. *P < 0.05, **P < 0.01 versus vehicle control; 1-way ANOVA followed by post hoc Student-Newman-Keuls test; *n* = 27 *SHP-1*^fl/fl^ vehicle, 14 *SHP-1*^fl/fl^ PD-L1, 23 *Na_V_1.8-Cre* vehicle, and 15 *Na_V_1.8-Cre* PD-L1 (cells). (**F**) KO of SHP-1 in Na_V_1.8^+^ neurons was confirmed by intracellular Lucifer dye injection and immunocytochemistry staining (Na_V_1.8 and SHP-1) after recording.

**Figure 6:**
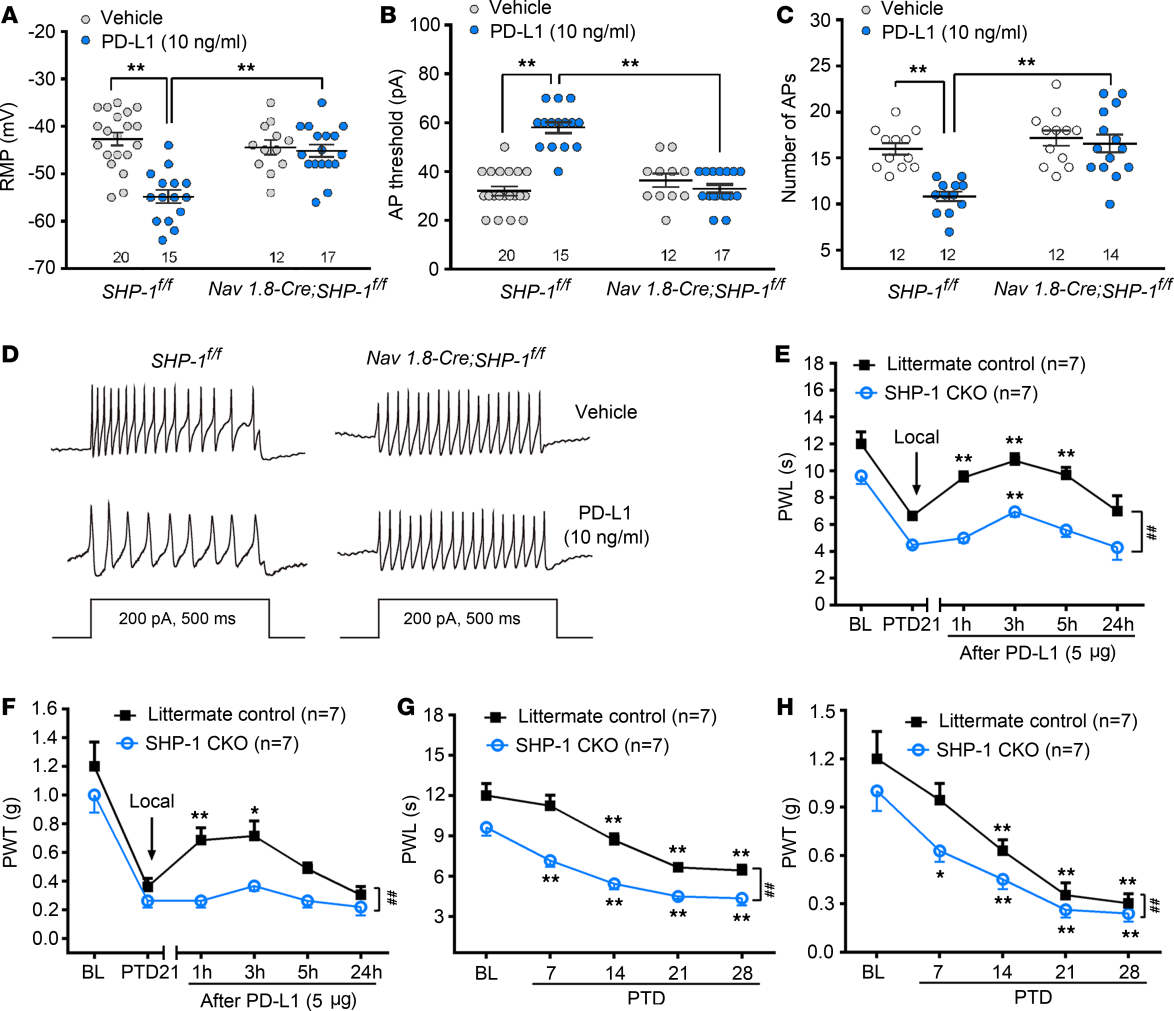
CKO of SHP-1 in Na_V_1.8-Cre DRG neurons attenuates the excitability of DRG neurons and blocks PD-L1–induced analgesic effect in bone cancer mice. (**A**–**C**) PD-L1 decreases resting membrane potential (RMP, **A**), increases action potential (AP) threshold (**B**), and reduces AP firing frequencies (**C**) in SHP-1^+/+^ DRG neurons of littermate control mice but not in SHP-1^–/–^ neurons of CKO mice. **P < 0.01; 1-way ANOVA followed by post hoc Student-Newman-Keuls test; *n* = 20 *SHP-1*^fl/fl^ vehicle, 15 *SHP-1*^fl/fl^ PD-L1, 12 *Na_V_1.8-Cre* vehicle, and 17 *Na_V_1.8-Cre* PD-L1 (cells). (**D**) Traces of APs in DRG neurons treated with vehicle (upper) or PD-L1 (10 ng/mL, bottom) in SHP-1^+/+^ DRG neurons of littermate control (left) and SHP-1^–/–^ neurons of CKO (right) mice. (**E** and **F**) The inhibition of PD-L1 on bone cancer–induced hyperalgesia (**E**) and allodynia (**F**) is lower in SHP-1–CKO mice than that in littermate controls. *P < 0.05, **P < 0.01 versus before PD-L1 injection on PTD 21; ^##^*P* < 0.01 versus littermate control mice; 2-way RM ANOVA followed by post hoc Student-Newman-Keuls test; *n* = 7 control and 7 SHP-1–CKO (mice). (**G** and **H**) SHP-1 CKO aggravates the bone cancer–induced thermal hyperalgesia (**G**) and mechanical allodynia (**H**). *P < 0.05, **P < 0.01 versus baseline; ^##^*P* < 0.01 versus littermate controls; 2-way RM ANOVA followed by post hoc Student-Newman-Keuls test; *n* = 7 control and 7 SHP-1–CKO (mice).

Na_V_1.8 is a voltage-gated sodium channel expressed only in a subset of sensory neurons of which more than 85% are nociceptors (34). We generated SHP-1–conditional KO (SHP-1–CKO) (35) mice by crossing B6.129P2-*Ptpn6^tm1Rsky^*/J (*SHP-1*^fl/fl^) mice with Tg(Scn10a-cre)1Rkun (*Na_V_1.8-Cre*) mice (36) (Figure 5A), leading to specific deletion of SHP-1 in nociceptive neurons as well as some low-threshold A-fiber neurons (36, 37). As shown in Figure 5B, there was no double staining signal of SHP-1 and Na_V_1.8 in DRG neurons from SHP-1–CKO mice. Western blot also showed a significant reduction of SHP-1 in SHP-1–CKO DRG neurons compared with littermate controls (Figure 5C). In acutely isolated neurons, whole-cell patch recordings showed that TRPV1 currents significantly enlarged in SHP-1–CKO mice compared with littermate controls (*SHP-1*^fl/fl^ mice), whereas PD-L1–induced inhibition on TRPV1 currents was abolished in SHP-1–CKO neurons (Figure 5, D–F), further confirming that SHP-1 mediated the modulation of PD-L1 on TRPV1. We then compared PD-L1’s effect on DRG neuronal excitability in SHP-1–CKO (*Na_V_1.8-Cre SHP-1*^fl/fl^) and littermate control (*SHP-1*^fl/fl^) mice. As shown in Figure 6, pretreatment of PD-L1 (10 ng/mL) for 30 minutes significantly decreased the resting membrane potential level, elevated action potential threshold, and reduced action potential firing frequency in DRG small-diameter neurons of littermate control mice (*SHP-1*^fl/fl^), which disappeared completely in CKO mice (Figure 6, A–D). Consistently, the analgesic effect of PD-L1 on bone cancer pain was weakened (allodynia) or eliminated (hyperalgesia) in SHP-1–CKO mice (Figure 6, E and F, 2-way RM ANOVA, treatments: *F*_[1, 12]_ = 97.77, *P* < 0.001 for PWL; *F*_[1, 12]_ = 20.52, *P* < 0.001 for PWT). We also compared development of bone cancer pain between littermate control and SHP-1–CKO mice (*Na_V_1.8-Cre SHP-1*^fl/fl^). Conditional KO of SHP-1 in Na_V_1.8^+^ DRG neurons promoted the earlier appearance (PTD 7) of cancer-induced thermal hyperalgesia and mechanical allodynia, suggesting a facilitation of bone cancer pain in SHP-1–CKO mice (Figure 6, G and H). Two-way RM ANOVA analysis revealed a significant intergroup difference (PWL, *F*_[1, 12]_ = 63.35, *P* < 0.001; PWT, *F*_[1, 12]_ = 8.14, *P* < 0.01).

## Discussion

Cumulative evidence shows that tumors and surrounding tissue cells release various algogenic substances, including protons, proteases, endothelins, vascular endothelial growth factor (VEGF), nerve growth factor (NGF), and transforming growth factor–β (TGF-β), in the tumor microenvironment to sensitize and injure primary sensory neurons (10, 14, 38–41). However, most cancer patients do not have pain in the early stages of disease (42). It is reasonable to speculate that the tumor microenvironment may produce not only pain factors but also analgesic factors at the same time. A collaborative study from Ji’s and our laboratories demonstrated that cancers such as melanoma produce the antinociceptive mediator PD-L1 to suppress pain via its receptor, PD-1, expressed on DRG neurons (15). PD-1/PD-L1 signaling in the trigeminal ganglia was also reported to be involved in acute nitroglycerin-induced hyperalgesia (43). In the present study we further provided several lines of evidence to support the analgesic effect of PD-L1 in LLC cell–induced mouse bone cancer pain. In particular, selective KO of SHP-1 in DRG Na_V_1.8^+^ neurons significantly attenuated the inhibitory effects of PD-L1 on TRPV1 current and bone cancer pain, suggesting that SHP-1–inhibited TRPV1 in DRG neurons contributes to PD-L1’s analgesic effect.

### PD-L1 alleviates bone cancer pain via suppressing TRPV1 function.

Previous studies revealed that following tumor infiltration TRPV1 expression increases in the DRG and contributes to bone cancer pain both in mice and in rats (9, 10). In rat advanced bone cancer, a robustly enhanced TRPV1 current and decreased desensitization rate to repetitive capsaicin application were observed in DRG neurons (10). Consistently, in a mouse bone cancer model, we observed a significant increase in TRPV1 protein level in DRG tissues from PTD 14 to 28. The dose-response curve of TRPV1 currents was left-shifted on PTD 21. Furthermore, TRPV1-KO mice failed to develop thermal hyperalgesia in the tumor-affected limb (Figure 3), suggesting that increased TRPV1 expression and TRPV1 sensitivity contribute to bone cancer–induced pain hypersensitivity. We have now shown that PD-L1 dose-dependently inhibited TRPV1 currents and capsaicin-induced [Ca^2+^]_i_ increase. PD-L1–induced analgesia on bone cancer pain only occurred in WT but not in TRPV1-KO mice. It is well known that PD-L1 suppresses immunity via interaction with its receptor, PD-1, on immune cells (16, 44, 45). Our previous (15) and current studies demonstrated that PD-1 expressed not only by immune cells but also DRG neurons, including TRPV1-positive DRG neurons. Thus, it is plausible that the analgesic effect of PD-L1 on bone cancer pain is achieved by inhibiting TRPV1 via PD-1 expressed by DRG neurons.

Three issues should be addressed. First, although our results and recent studies showed that PD-1 (encoded by Pdcd1) mRNA and protein were expressed in primary sensory neurons and were involved in acute and chronic pain as well as opioid-induced hyperalgesia (15, 43, 46), single-cell RNA-Seq failed to detect Pdcd1 mRNA expression (47–50) in the DRG neurons. A possible explanation is that single-cell RNA-Seq can only detect a limited transcriptome; some low- to medium-expressed but important genes in sensory neurons might be missed. For example, *Shank3* and *Vegfrs* have not been detected by single-cell analysis but are expressed in DRG neurons (41, 47). Second, despite conflicting reports regarding the involvement of TRPV1 in mechanical hypersensitivity evoked by inflammatory and neuropathic pain (11, 51, 52), we observed in the present study that mechanical allodynia in cancer-bearing mice is partially attenuated in TRPV1-KO mice, and the antiallodynia of PD-L1 was eliminated in TRPV1-KO mice. As a support, Fang et al. revealed that TRPV1 antagonist CPZ significantly blocks bone cancer–induced mechanical allodynia in rats (53). Consistently, carrageenan-induced mechanical allodynia (54), experimental autoimmune prostatitis–induced pelvic tactile allodynia (55), and chronic morphine-induced tactile hypersensitivity are nearly lost in TRPV1-KO mice (56). It has been reported that TRPV1 is expressed not only in small neurons but also in medium to large neurons, and upregulated TRPV1 in bone cancer mouse DRG neurons mainly occur in calcitonin gene–related peptide–positive peptidergic neurons and neurofilament 200–positive myelinated neurons (9), which have previously been implicated in mechanical allodynia (57–59). Third, although increased PD-L1 expression in tumor-bearing bone was detected at all time points in this study, the endogenous PD-L1 masked bone cancer–induced pain only in the early phase. We found that with blockade of PD-1 with nivolumab (a humanized IgG4 monoclonal antibody targeting PD-1) on PTD 7, when the LLC cell inoculation–induced pain hypersensitivity had not developed, both hyperalgesia and allodynia were evoked in the tumor-affected limb. One possible explanation is that increased PD-L1 may not be sufficient to overcome the exaggerated pain caused by various algogenic substances released during advanced bone cancer, such as VEGF (41), TGF-β_1_ (10), and NGF (40). A recent study demonstrated that acute i.v. injection of anti–PD-1 antibody nivolumab (10 mg/kg) evoked rapid increases in mechanical allodynia and thermal hyperalgesia on days 3 and 7 after LLC cell inoculation, while chronic PD-L1 treatment promoted osteoclastogenesis, and chronic repeated nivolumab (i.v.) even attenuated bone cancer–induced allodynia and hyperalgesia by suppressing osteoclastogenesis, suggesting endogenous PD-L1–mediated antinociception may be transient in the early phase of bone cancer (60).

### Inhibition of TRPV1 by SHP-1 is a key event in PD-L1 analgesia.

How does PD-L1 modulate TRPV1 function? Activation of PD-1 recruits the tyrosine phosphatases SHP-1 and SHP-2 to mediate biological actions of PD-L1 in immune cells (18, 19). Primary nociceptors and immune cells have some similarities; for example, nociceptors express major immune modulators, including cytokines, chemokines, and TLRs (61, 62). In the present study, we found that SHP-1 was also expressed in DRG neurons and colocalized with PD-1. Coimmunoprecipitation displayed interaction of SHP-1 and PD-1 proteins. Following the bone cancer development, DRG p–SHP-1 was upregulated in a similar time course to PD-L1 in tumor-bearing bone. PD-L1 treatment significantly upregulated p–SHP-1 in cultured DRG neurons, especially TRPV1-positive neurons (Figure 4). In SHP-1–deficient DRG neurons, PD-L1–induced decrease in excitability was prevented. Conditional KO (35) of SHP-1 in DRG nociceptors (*Na_V_1.8-Cre Shp-1*^fl/fl^) robustly blocked antihyperalgesia and antiallodynia of PD-L1 in bone cancer mice (Figure 6). Thus, in DRG neurons SHP-1 as a downstream signaling event of PD-1 activation contributes to PD-L1–evoked analgesia.

SHP-1 is a widely expressed cytosolic protein tyrosine phosphatase (63–65). Its importance is underscored by a myriad of its substrates and its dephosphorylation correlating with cancer (66) and pain (67, 68). Tyrosine phosphorylation was also reported to be important for TRPV1 activation (20, 69, 70). Nonspecific PTP inhibitor increases the tyrosine phosphorylation of TRPV1 in HEK293 cells (69). The increase in tyrosine phosphorylation of TRPV1 in DRG neurons was also induced by inhibition of SHP-1, and SHP-1 can alleviate CFA-induced inflammatory pain by dephosphorylating TRPV1 and suppressing TRPV1 expression in DRG neurons (21). In the current study, we further demonstrated that inhibition or KO of SHP-1 markedly sensitized TRPV1 currents in DRG small-diameter neurons. I.pl. injection of SHP-1 inhibitor PTPiIII directly induced hyperalgesia, allodynia, and spontaneous nociception-like licking/lifting behaviors in WT but not in TRPV1-KO mice (Figure 4). We also detected a decreased basal pain threshold and exaggerated thermal hyperalgesia and mechanical allodynia following bone cancer development in CKO SHP-1 mice (Figure 6). Moreover, we found in SHP-1–deficient DRG neurons the suppression of PD-L1 on TRPV1 currents was completely abolished (Figure 5), indicating that PD-L1’s inhibition on TRPV1 was mediated by SHP-1. Our results also suggest that SHP-1 in sensory neurons is an endogenous pain inhibitor and delays the development of cancer-induced bone pain via suppressing TRPV1 function.

It is worth mentioning that SHP-1 inhibitor not only augmented TRPV1 currents but also blocked repeated capsaicin-induced desensitization of TRPV1 (Figure 4). It is well known that TRPV1 functions can be sensitized or desensitized. The sensitization occurs through protein kinase–dependent phosphorylation and subsequently depolarizes the neurons and increases excitability. Desensitization can be mediated via protein phosphatase-induced dephosphorylation, by calmodulin interaction with TRPV1 in a Ca^2+^-dependent manner, or by inositol 1,4,5-trisphosphate–induced calcium release from intracellular stores (35, 71). It has been demonstrated that dephosphorylation of TRPV1 by protein phosphatase 2B is a critical mechanism that leads to desensitization of the channel (72, 73). Nonspecific inhibition of PTPs increases the tyrosine phosphorylation of TRPV1 and sensitizes TRPV1 (20). Inhibition of SHP-1, a nonreceptor PTP expressed in the cytoplasm, amplified repeated capsaicin responses, suggesting that SHP-1 is also a key phosphatase for dephosphorylation of TRPV1.

In conclusion, our previous study demonstrated that PD-L1, as a neuromodulator, plays a critical role in inhibition of baseline pain, acute inflammatory pain, and chronic neuropathic pain. Here, we further found that PD-L1/PD-1 signaling inhibited TRPV1 activity via SHP-1 in primary sensory neurons and thereby suppressed and delayed the development of bone cancer pain in mice. Our studies highlight the possibility of cancer cell–derived pain inhibitors for developing early diagnosis markers and new analgesic drugs.

## Methods

### Animals

Adult (8–10 weeks) C57BL/6J WT, TRPV1-KO (003770, Jackson Laboratory, San Mateo, California, USA), SHP-1^fl/fl^ mice (008336, Jackson Laboratory), Ai 14-reporter (B6.Cg-Gt(ROSA)26Sor^tm14(CAG-tdTomato)Hze/^J, 007914, Jackson Laboratory), and *Na_V_1.8-Cre* (gift from Rohini Kuner, Heidelberg University, Heidelberg, Germany) mice were used in this study (male C57BL/6J, both male and female transgenic strains). SHP-1–CKO mice were generated by mating *Na_V_1.8-Cre SHP-1*^fl/fl^ mice. All animals were housed in cages under a 12-hour light/12-hour dark cycle with food and water available ad libitum. Animals were randomly assigned to each group. All the following behavioral testing, electrophysiological recording, and quantification of Western blot experiments described herein were performed by experimenters who were blind with respect to the treatments.

### Reagents

Active mouse PD-L1 protein fragment (catalog ab216261) and human IgG4 control (catalog ab90286) were purchased from Abcam (Cambridge, Massachusetts, USA). Anti–PD-1 antibody nivolumab (Opdivo) was purchased from Bristol-Myers Squibb (New York, New York, USA). SHP-1 inhibitor SSG (catalog 567565) and PTPiIII (catalog 540210) were from Calbiochem (Darmstadt, Germany). Capsaicin (catalog M2028) was purchased from MilliporeSigma (St. Louis, Missouri, USA).

### PD-1–specific hairpin RNA and virus preparation

The mouse *Pd1*-specific hairpin RNA (shRNA) sequence was designed based on the work of Zhao and coworkers (74, 75); PD-1–shRNA (GATCCGGGTTTGAGCCAACCCGTCCAGTTCAAGAGACTGGACGGGTTGGCTCAAACCTTTTTTGGAAA) or sh-control RNA (the scrambled sequence) was synthesized by Shanghai GeneChem Co., Ltd (Shanghai, China). The recombinant virus coexpressing enhanced green fluorescent protein was packaged using a Lentivector Expression system kit (Shanghai GeneChem Co., Ltd).

### Drug administration

For local injection, PD-L1 (2 μg or 5 μg in 100 μL normal saline, NS) was delivered into local deep tissue around the tumor bone with a 30-gauge needle. For i.v. injection, anti–PD-1 antibody (nivolumab, 10 mg/kg in 100 μL NS) or control antibody (human IgG4) was administered into the tail vein of mice. For i.pl. injection, drugs were injected in 20 μL NS using a Hamilton microsyringe with a 30-gauge needle. For LP injection, drugs were delivered into the spinal space via an LP performed with a 30-gauge needle between the L_5_ and L_6_ vertebrae under isoflurane anesthesia (3% for induction and 1.5% for maintenance). Mice that showed any surgery-related neurological deficits were excluded from the experiment.

### Bone histology

Mice were deeply anesthetized with overdose of sodium pentobarbital and transcardially perfused with 300 mL of 0.9% NS followed by 300 mL of 4% paraformaldehyde. Bilateral femur bones were removed and decalcified in decalcifying solution for 24 hours. The bones were rinsed and dehydrated and then embedded in paraffin, cut into 7 μm cross sections using a rotary microtome (Reichert-Jung 820, Cambridge Instruments), and stained with hematoxylin and eosin to visualize the extent of tumor infiltration and bone destruction.

### ISH using RNAscope

ISH was performed using a commercial probe for mouse PD-1 (Advanced Cell Diagnostics, 416781, Newark, California, USA) and the RNAscope 2.5 High Definition Brown Assay according to the manufacturer’s protocol. The DRG images were captured using a Leica Microsystems microscope (Wetzlar, Germany).

### RT-PCR

Mouse DRG tissues or cells were collected and homogenized in TRIzol reagent (Invitrogen, Thermo Fisher Scientific, Carlsbad, California, USA). Total RNA was extracted following the manufacturer’s protocol. RNAs (0.5–1 g) were reverse-transcribed using the SuperScript III reverse transcriptase (Invitrogen, Thermo Fisher Scientific). The sequences of primers were as follows: PD-1, forward: 5′-TGCTCAACAAGTATGTCAGAGG-3′, reverse: 5′-ACACTAGGGACAGGTGCTGC-3′; GAPDH, forward: 5′‑AGGTCGGTGTGAACGGATTTG‑3′, reverse: 5′‑TGTAGACCATGTAGTTGAGGTCA‑3′; TRPV1, WT forward: 5′‑TGGCTCATATTTGCCTTCAG‑3′, mutant forward: 5′‑TAAAGCGCATGCTCCAGACT‑3′, common reverse: 5′‑CAGCCCTAGGAGTTGATGGA‑3′ (Sangong Biotech, Shanghai, China).

### Immunohistochemistry

After appropriate survival times, mice were deeply anesthetized and perfused with warm NS followed with 4% cold paraformaldehyde in 0.1 M PBS. DRG tissues (L_3_–L_5_ segments) were removed and postfixed in the same fixative for 2 hours at 4°C and then immersed in a 10%–30% gradient of sucrose in PBS for 24–48 hours at 4°C for cryoprotection. DRG sections (14 μm) were cut by a freezing microtome (Leica Microsystems). Control and treated DRG sections were mounted on the same slides and processed under the same conditions. The sections were blocked with 10% donkey serum in PBS, pH 7.4, with 0.3% Triton X-100 for 2 hours at room temperature (RT) and incubated for 24–36 hours at 4°C with the following primary antibodies: goat anti–PD-1 (1:500, R&D Systems, Bio-Techne, catalog AF1021, Minneapolis, Minnesota, USA), rabbit anti–Substance P (1:2000, Peninsula Laboratories, catalog T-4107.0050, San Carlos, California, USA), rabbit anti–SHP-1 (1:500, GeneTex, catalog GTX102864, Irvine, California, USA), goat anti-Nav1.8 (1:1000, Abgent, catalog AF3512a, San Diego, California, USA), guinea pig anti-TRPV1 (1:1000, Alomone Labs, catalog AGP-118, Jerusalem, Israel). After three 15-minute rinses in PBS, the sections were incubated with a mixture of Alexa Fluor 488– or Alexa Fluor 546–conjugated secondary antibodies (1:200, catalog A-11055/A10036, Invitrogen, Thermo Fisher Scientific), or IB4-Alexa Fluor 488 (1:200, catalog A21206, Invitrogen, Thermo Fisher Scientific) for 2 hours at RT. The specificity of immunostaining and primary antibodies was verified by omitting the primary antibodies, by testing KO and CKO mice or shRNA-knockdown cells, and by in situ hybridization. The stained sections were coverslipped and examined by a confocal laser scanning microscope (FV1000; Olympus, Tokyo, Japan).

### Western blotting

Mice were deeply anesthetized, and the L_3_–L_5_ DRG tissues and femurs (near the metaphysis) from bone cancer and control mice were collected and then homogenized in the lysis buffer (12.5 μL/mg tissue) containing a mixture of protease inhibitors and phenylmethylsulfonyl fluoride (Roche Diagnostics, Basel, Switzerland). The protein concentrations of the lysate were measured using a BCA Protein Assay kit (Pierce, Thermo Fisher Scientific, Rockford, Illinois, USA). Equal amounts of protein samples were loaded and separated in 10% SDS-PAGE and then transferred to PVDF membranes (MilliporeSigma, Billerica, Massachusetts, USA). The membranes were blocked with 5% nonfat milk in Tris-buffered saline (pH 7.5) with 0.1% Tween-20 for 2 hours at room temperature and incubated overnight at 4°C with primary antibodies, followed by HRP-conjugated secondary antibodies (1:2000, Santa Cruz Biotechnology) for 2 hours at room temperature. For loading control, the blots were probed with GAPDH antibody. Signals were visualized using enhanced chemiluminescence (Pierce, Thermo Fisher Scientific) and captured by ChemiDoc XRS system (Bio-Rad, Hercules, California, USA). We used the following primary antibodies: rabbit anti–PD-L1 (1:1000, GeneTex, catalog GTX31308), goat anti–PD-1 (1:1000, R&D Systems, Bio-Techne, catalog AF1021), rabbit anti-TRPV1 (1:1000, catalog ACC-030, Alomone Labs), rabbit anti–p–SHP-1 (1:1000, catalog 8849, Cell Signaling Technology, Danvers, Massachusetts, USA), rabbit anti–SHP-1 (1:1000, catalog GTX102864, GeneTex). HRP-conjugated secondary antibody used included donkey anti–mouse IgG–HRP antibody (1:2000, catalog sc-2318, Santa Cruz Biotechnology); donkey anti–goat IgG–HRP antibody (1:2000, catalog sc-2020, Santa Cruz Biotechnology); donkey anti–rabbit IgG–HRP antibody (1:2000, catalog sc-2077, Santa Cruz Biotechnology). All Western blot analysis was performed 3 to 4 times, and consistent results were obtained. A Bio-Rad image analysis system was then used to measure the integrated optic density of the specific bands.

### Preparation of acutely isolated DRG neurons

Mice were anesthetized with isoflurane and then rapidly decapitated. The DRG tissues from spinal L_3_–L_5_ segments were removed and immediately transferred onto DMEM (Invitrogen, Thermo Fisher Scientific) on ice. The ganglia were minced with fine spring scissors and treated with collagenase (type IA, 2.67 mg/mL, MilliporeSigma) and trypsin (type I, 1 mg/mL, MilliporeSigma) in DMEM at 37°C for 30 minutes. After washing with a standard external solution, the ganglia were then gently triturated using fine fire-polished Pasteur pipettes. To conduct electrophysiology experiments, the isolated DRG neurons were plated onto glass coverslips in 3.5 cm culture dishes and incubated with a standard external solution containing (in mM) 140 NaCl, 5 KCl, 2.5 CaCl_2_, 1 MgCl_2_, 10 HEPES, and 10 glucose, pH 7.4, at RT for at least 2 hours.

### Whole-cell patch clamp recordings

Whole-cell voltage clamp and current clamp recordings of DRG neurons were performed at RT with an Axon patch 700B amplifier (Molecular Devices, Thermo Fisher Scientific, San Jose, California, USA) as previously described (10). All of the recordings were performed in small-diameter (<25 μm) DRG neurons and were made 2–6 hours after plating, with resting membrane potentials less than –50 mV. Microelectrodes (N51A borosilicate glass, Sutter Instruments, Novato, California, USA) with a resistance of 3–6 MΩ were pulled using a P97 puller (Sutter Instruments). The pipette solution contained (in mM) 140 KCl, 1 MgCl_2_, 5 EGTA, 3 Na_2_ATP, 0.4 NaGTP, and 10 HEPES, pH 7.2. Seals (1–10 G) between the electrode and the cells were established. After the whole-cell configuration was established, the cell membrane capacitance and series resistance were compensated for (>80%), and leak currents were subtracted using the online P/4 protocol. The data were low-pass-filtered at 2 kHz and sampled at 10 kHz. Capsaicin-induced TRPV1 currents were recorded in voltage-clamp mode, with the membrane potential held at –65 mV. Action potentials were elicited by injecting a depolarizing current (200 pA, 500 ms). Drugs were applied using a DVD-8VC super fusion application system (ALA Scientific Instruments, Farmingdale, New York, USA). Only 1 recording was performed on each dish to ensure that data were not obtained from cells that had been inadvertently exposed to other test treatments. Per animal 2 or 3 cells were studied to ensure the data were from different animals. The pClamp10 (Axon Instruments, San Jose, California, USA) software was used during experiments and analysis.

### Calcium imaging

The acutely isolated DRG neurons were loaded with 1 μM fura-2 AM (DoJinDo Laboratories, Kumamoto, Japan) for 1 hour and were then washed and incubated with standard external solution. The entire process was protected from light. The neurons were observed on an inverted microscope (Olympus IX51) with a ×40 UV fluor oil immersion objective lens. The fluorescence of the individual neurons was recorded by a cooled charge-coupled device camera (Hamamatsu Photonics, Hamamatsu City, Japan), with a 1 Hz alternating wavelength, time scanning with excitation wavelengths of 340 and 380 nm, and an emission wavelength of 510 nm (Lambda DG5, Sutter Instruments). Images were captured every 1 second. Digitized images were acquired and analyzed by SimplePCI (Compix, Sewickley, Pennsylvania, USA). The ratio of the fluorescence at the 2 excitation wavelengths was represented to estimate the changes in the [Ca^2+^]_i_.

### DRG neuron culture and transfections

Acutely isolated DRG cells in bacteria-free environment were placed on glass coverslips coated with poly-d-lysine and grown in a Neurobasal defined medium (10% FBS, 2% B27 supplement, 0.5 mM l-glutamine, 100 U/mL penicillin, and 100 U/mL streptomycin) at 37°C with 95% air/5% CO_2_. Cultured neurons were transfected with shRNA-lentivirus 3 days before follow-up experiments.

### Culture of murine LLC cells

LLC cells, which originated as a spontaneous carcinoma of the lung in C57BL/6 mice, were purchased from Cell Bank of Type Culture Collection of Chinese Academy of Sciences (Shanghai, China). LLC cells were grown in DMEM (Gibco, Thermo Fisher Scientific, New York, New York, USA) containing 10% heat-inactivated fetal bovine serum (Gibco, Thermo Fisher Scientific), penicillin (100 U/mL), and streptomycin (100 U/mL) (Gibco, Thermo Fisher Scientific) in 95% air/5% CO_2_ at 37°C.

### Mouse bone cancer model

To inoculate LLC cells, mice were anesthetized with sodium pentobarbital (50 mg/kg, i.p.). The left leg was then shaved, and the skin was disinfected with iodine tincture and 75% ethanol. A 27-gauge needle was inserted at the site of the intercondylar eminence of the left femur and was then replaced with a 10 μL microinjection syringe containing a 4 μL suspension of tumor cells (1.0 × 10^6^). The contents of the syringe were slowly injected into the femur cavity. To prevent the leakage of cells outside the bone, the injection site was sealed with bone wax. For the sham group (controls), 4 μL of PBS was injected instead of carcinoma cells into the femur. At the end of the experiment, radiological, postmortem, and histological evaluations were performed. Mice that showed no obvious tumor growth and bone destruction after the inoculation with tumor cells were excluded from the experiments.

### Behavioral experiments

#### von Frey test for mechanical pain.

Animals were habituated to the testing environment daily for at least 3 days before testing. The room temperature and humidity remained stable for all experiments. Mechanical allodynia was assessed by measuring PWTs in response to a calibrated series of von Frey hairs (0.16–4.0 g, Stoelting Company, Wood Dale, Illinois, USA). Each mouse was placed in a chamber (9 cm × 9 cm × 4.5 cm) on an elevated metal mesh floor. Mice were allowed to acclimate for approximately 30 minutes. A series of von Frey filament stimuli (0.16, 0.4, 0.6, 1.0, 1.4, 2.0, 4.0 g) were delivered to the central region of the plantar surface of the hind paw with increasing bending force until the mouse withdrew the foot. Each filament was applied 5 times and each time maintained for 2 seconds with 15-second intervals. Once the hind paw was withdrawal from a particular hair 3 out of the 5 consecutive applications, the value of the filament in grams was considered the PWT.

#### Hargreaves test for thermal pain.

Thermal hyperalgesia was assessed by measuring the PWLs in response to a radiant heat source (IITC Life Science Instruments, Woodland Hills, California, USA). Mice were placed individually into Plexiglas chambers on an elevated glass surface and allowed to acclimate for 30 minutes. The heat source was turned off when the mouse lifted its foot, allowing for measurement of the time from the onset of radiant heat application to the withdrawal of the hind paw. This time was defined as the PWL. The heat was maintained at a constant intensity, which produced a stable PWL of 10–12 seconds in naive mice. A 15-second cutoff was used to prevent tissue damage in the absence of a response.

#### Spontaneous pain.

To assess spontaneous pain, each mouse was placed in a raised Plexiglas chamber and allowed to acclimate for 1 hour before testing. The number of times the hind paw ipsilateral to the treated paw flinched and of episodes of licking were counted during a 10-minute observation period.

#### CatWalk gait analysis.

The CatWalk system (XT, Noldus Information Technology, Wageningen, the Netherlands) was used for the quantitative assessment of gait parameter and footfalls in rodents. Gait analysis has proved a reliable method for measuring pain-associated behaviors, based on the voluntary movement of rodents in an enclosed walkway (24, 76, 77). Briefly, the mouse was placed in the open end of the enclosed glass platform in a darkened room with a red ceiling light-emitting diode light and allowed to walk voluntarily through the walkway. While the mouse walked across the glass floor, a high-speed camera positioned underneath the apparatus captured images of the illuminated area of each paw and transferred the data to the gait analysis software (CatWalk XT, version 10.0; Noldus Information Technology). A minimum of 3 serial step cycles, or complete passes through the tunnel, were gathered as valid data. In this study, 3 available parameters were identified to evaluate dynamic behaviors associated with bone cancer pain: (a) print area (in square centimeters) represents the surface of the complete print of a paw; (b) stand (in seconds) is the duration of ground contact for a single paw; (c) swing (in seconds) is the duration of no hind paw contact with the glass plate. Data were calculated as the percentage of ipsilateral/contralateral hind paw.

#### Conditioned place preference.

The place-conditioning apparatus and CPP procedure were as described previously with slight modifications (78, 79). The place-conditioning apparatus consists of 3 opaque acrylic compartments (1 neutral chamber and 2 conditioning chambers with distinct olfactory and visual cues). The experimental process consists of 3 distinct sessions: a preconditioning session, a conditioning session, and a test (postconditioning) session. All mice underwent a 3-day preconditioning habituation and animal behavior was video recorded. Day 1 was the preconditioning day. At the beginning, a mouse was placed in the neutral compartment. After habituating for 2 minutes, the entrance to each conditioning compartment was opened. When the mouse entered any conditioning compartment, the door connecting the neutral and conditioning compartment was closed. The mouse was allowed to explore the 2 conditioning compartments freely for 10 minutes. A timer automatically recorded the time spent in each of the compartments in a blind manner. Mice that spent more than 80% (480 seconds) on one side on that day were eliminated from the subsequent experiments. Day 2 was the conditioning day. On this day, all doors were closed. The mouse received no treatment in the morning and was randomly confined to one of the conditioning compartments for 60 minutes. After at least 3 hours, in the afternoon, CPP training mice were given a local injection of PD-L1 (2 μg) (or vehicle as control) and then restrained in the other conditioning compartment for 60 minutes. Day 3 was the postconditioning day. The procedure was the same as day 1, and the time animals spent in each compartment was measured. Analgesic effect was determined by whether time spent in the PD-L1–paired compartment grew longer.

### Statistics

All data were presented as mean ± SEM and analyzed using GraphPad Prism 7.0 software (San Diego, California, USA). No statistical power calculation was conducted before the study. The sample sizes were based on our previous knowledge and experience with this design. There were no missing data. All data from different groups were verified for normality and homogeneity of variance using Kolmogorov-Smirnov and Brown-Forsythe tests before analysis. Behavioral data were analyzed using Student’s *t* test when comparing 2 groups or 1-way or 2-way RM ANOVA followed by post hoc Bonferroni’s multiple-comparisons test when comparing more than 2 groups. Electrophysiological recording and Western blot data were compared using Student’s *t* test (2 groups) and 1-way ANOVA followed by post hoc Dunnett’s test (more than 2 groups). No data were excluded from statistical analyses due to outlier status. All the hypothesis testing was 2-tailed with *P* value less than 0.05 considered statistically significant.

### Study approval

All animal procedures were approved by the IACUC of Fudan University (permit SYXK2009-0082) and followed the policies issued by the guidelines for pain research of the International Association for the Study of Pain.

## Author contributions

BLL and QLC developed the project; performed electrophysiological, immunohistochemical, in situ hybridization, and behavioral experiments; and prepared final figures. XZ and HZL conducted mouse tumor model and TRPV1-KO mouse experiments. YQZ supervised the project. YQZ and BLL wrote the paper.

## Supplementary Material

supplemental data

## Version 1 09/22/2020

In-Press Preview

## Version 2 10/15/2020

Electronic publication
