# Supplementary material for: Inhibition of TRPV1 by SHP-1 in nociceptive primary sensory neurons is critical in PD-L1 analgesia
Source: JCI Insight. 2020 Oct 15;5(20):e137386. doi: 10.1172/jci.insight.137386 (PMC7605531; doi:10.1172/jci.insight.137386)
Supplement: supplemental data [file jciinsight-5-137386-s126.pdf]

Supplemental Information

Inhibition of TRPV1 by SHP-1 in nociceptive primary sensory neurons is critical in PD-L1 analgesia

Ben-Long Liu[1, §], Qi-Lai Cao[1, §], Xin Zhao[1], Hui-Zhu Liu[1], Yu-Qiu Zhang[1, 2]*

[1]State Key Laboratory of Medical Neurobiology and MOE Frontiers Center for Brain Science, Department of Translational Neuroscience, Jing'an District Centre Hospital of Shanghai, Institutes of Brain Science, Fudan University, Shanghai 200032, China.

[2]Institutes of Integrative Medicine, Fudan University, Shanghai 200032, China.

Authorship note:

[§]BLL and QLC share first authorship (they contributed equally to this work).

*Corresponding author

Inventory of Supplemental Information

Figure. S1. Related to Figure 2, Expression of PD-1 in the mouse DRG.

Figure S2. Related to Figure 3, Knockout of TRPV1 blocks bone cancer-induced pain-like behaviors.

Supplemental Figures

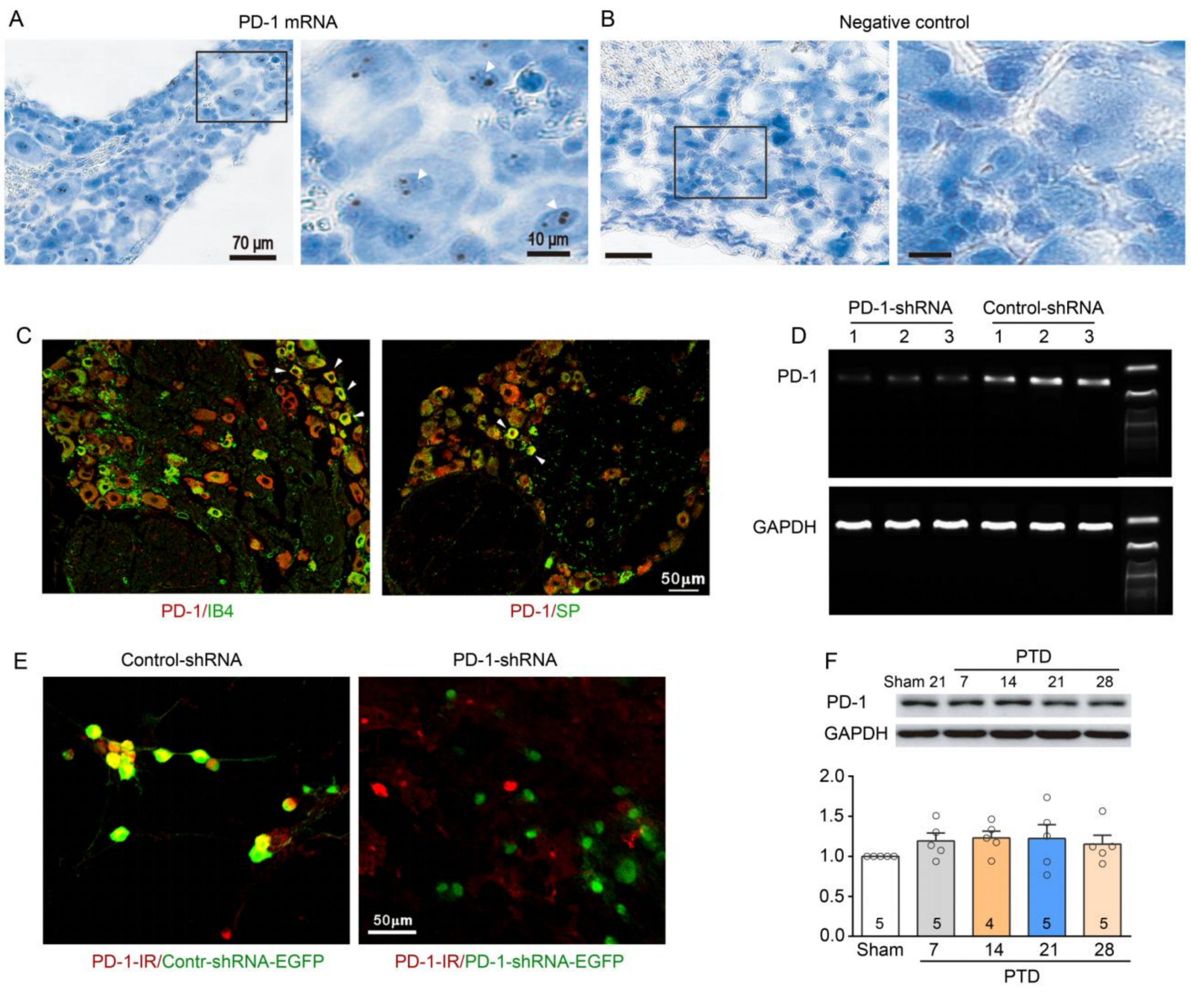

Figure. S1. Related to Figure 2, Expression of PD-1 in the mouse DRG. (A & B) RNAsope *in situ* hybridization (ISH) showing PD-1 mRNA expression in L_4 DRG of naive mice (A). B shows negative control. Scale bar: 70 µm (low magnification) and 10 µm (high magnification). (C) Double immunofluorescence staining reveals the expression of PD-1 in IB4 positive and SP positive neurons in L_4 DRG. Scale bar: 50 µm. (D) RT-PCR showing the efficiency of PD-1 knockdown. PD-1 mRNA level was decreased in cultured DRG neurons from three independent experiments at 3 days after transfection of PD-1 shRNA-lentivirus. GAPDH mRNA was used as internal control. (E) The specificity of PD-1 antibody was verified in PD-1 knockdown cells. Few PD-1 immunoreactivity (IR, red) colocalized with PD-1 shRNA-EGFP (right) in cultured DRG neurons. Scale bar: 50 µm. (F) Western blot analysis showing PD-1 level in the L3-L5 DRGs after tumor inoculation. n.s. no significant, one-way ANOVA; n=5, 5, 4, 5 and 5 (mice).

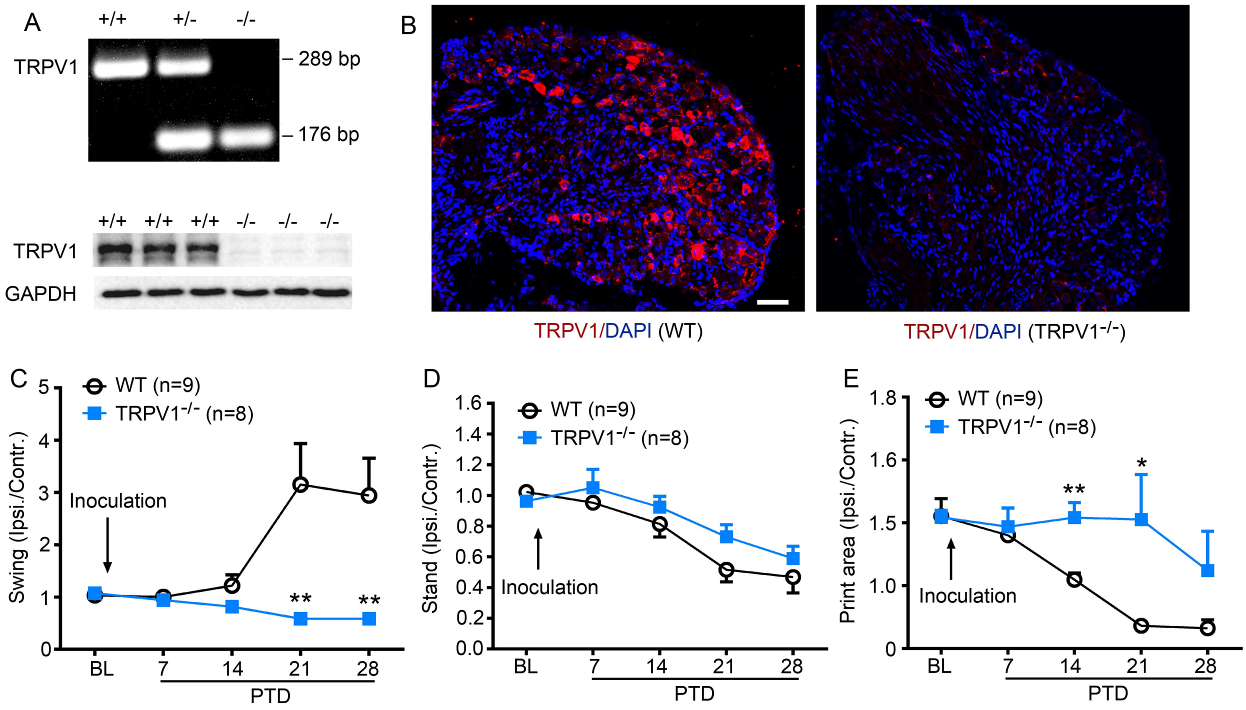

Figure S2. Related to Figure 3, Knockout of TRPV1 blocks bone cancer-induced pain-like behaviors. (A and B) TRPV1 knockout mice showing lost TRPV1 signal by RT-PCR, western blot and immunohistochemisty. (C–E) CatWalk gait analysis showing lack of pain-like behaviors in TRPV1 knockout mice after tumor inoculation. *$p<0.05$, **$p<0.01$ versus wild-type mice; two-way RM ANOVA followed by post hoc Student-Newmann-Keuls test; n=8 and 9 (mice).
